# SMASH: Screening Molecules Accurately on Small Hardware. Fast, user-friendly, enhanced with a machine learning virtual screening tool

**DOI:** 10.1007/s00894-026-06837-x

**Published:** 2026-07-04

**Authors:** Alfredo Suárez-Alonso, Leonardo D. Herrera-Zúñiga, Mayra Lozano-Espinosa, Abraham Giacoman-Martínez, Edgar F. Alarcón-Villaseñor, Julio C. Almanza-Pérez, Francisco J. Alarcón-Aguilar

**Affiliations:** 1 Laboratorio de Farmacología, Departamento de Ciencias de La Salud, Universidad Autónoma Metropolitana-Iztapalapa, Ciudad de México, México; 2 Departamento de Ciencias Naturales, Universidad Autónoma Metropolitana-Cuajimalpa, Ciudad de México, México; 3 HS Estudios Farmacoeconómicos S.A. de C.V., Iztapalapa, Ciudad de Mexico, México; 4 Departamento de Ciencias Naturales, SECIHTI-Universidad Autónoma Metropolitana-Cuajimalpa, Ciudad de México, México; 5 Centro de Investigación Biomédica del Sur, Instituto Mexicano del Seguro Social, Xochitepec, Morelos, México; 6 Residencia en Psiquiatría, Servicio de Psiquiatría, Centro Médico Nacional Hospital 20 de Noviembre. ISSSTE, Ciudad de México, México

**Keywords:** Virtual screening, Molecular docking, Machine learning, GPU computing, Open-source software, Drug discovery

## Abstract

**Context:**

In 2025, we released UAM-Ixachi to democratize, simplify, and accelerate molecular docking and virtual screening methods. It is a free, open-source, and user-friendly tool. Building on that work, we present SMASH, which features several upgrades: it predicts binding sites using machine learning, automatically determines titration states, utilizes a Graphics Process Unit for molecular docking, combines machine learning with energy scoring functions, and clusters data using the K-means algorithm. Tests demonstrated that the tool might handle large projects within a reasonable time using local computational resources. In automatic mode, the tool can accurately reproduce a high percentage of ligand poses from Protein Data Bank complexes via docking simulation. It also differentiated between the predictions of active and decoy ligands from a DUD-E data set in a reasonable amount of time. SMASH is freely available at https://smashreleases.z13.web.core.windows.net/

**Methods:**

We implemented several software solutions to prepare and execute molecular docking simulations: PDB2PQR, P2Rank, MGL Tools, OpenBabel, AutoDock-GPU, Vina-GPU, and SCORCH. The MMFF94, AD4, and Vina force fields are part of the implemented tools.

**Supplementary Information:**

The online version contains supplementary material available at 10.1007/s00894-026-06837-x.

## Introduction

The concept of large-scale compound screening dates to the mid-1990s, following the implementation of High-Throughput Screening. This robotic technique aims to automate the exhaustive search for promising compounds in biotechnology and pharmaceuticals by advancing assay miniaturization, compound synthesis and isolation, and the availability of molecular targets [1]. This can be pursued using exclusively computational methods.

Among the available methodologies, molecular docking is widely used for virtual screening. Docking methods are well-established tools for predicting molecules with therapeutic potential. One of the most widespread docking methods combines two algorithms: one for exhaustively searching for multiple docking poses within a protein region, and another for scoring these poses to identify promising molecules. In this field, there are solutions that, while offering user-friendly features (including support services), are usually commercial products that require expensive licensing and subscription fees. On the other hand, free and open-source tools, although powerful, can be challenging to learn and implement. Thus, the execution of virtual screening projects may be limited by the budget, computational simulation expertise, and the hardware available to researchers.

With recent technological advances, graphics processing units (GPUs) are becoming increasingly powerful, even those sold as integrated components in low-cost computers. Users may own computers with GPUs onboard without even realizing it, and these machines are likely capable of running molecular docking simulations more efficiently than central processing unit (CPU)-based methods. Of course, users with mid-range or high-end GPUs can exploit their hardware to run highly efficient simulations of pharmacological interest.

There is currently a rapid development in artificial intelligence (AI) with practical applications. In biology and medicine, there are complex systems that require analysis, for which AI applications can offer new possibilities. AI models demonstrate great versatility, addressing a wide range of problems, including predictions of protein folding, dopaminergic neuron interactions, drug design and optimization, and supporting clinical trial management, among other areas [2–4]. While computational predictive methods already represent a substantial improvement in terms of budget, time, and success rate in drug development, with AI support, the improvement can be greater.

We aim to develop tools that support the early stages of drug development, not only practical but also accessible to the broadest possible range of researchers. In 2025, UAM-Ixachi [5] was released, implementing several algorithms that automatically address various challenges in preparing and executing molecular docking simulations. In this work, several of the limitations of this algorithm have been addressed. SMASH is a new virtual screening tool that is faster, more user-friendly, and more accurate, and it can run on modest hardware.

## Methods

### Overall workflow, inputs, and computational requirements

SMASH was written in Python 3.11 and is composed of small pieces that work together with a bit of Bash scripting. The program was designed to enable people with varying levels of experience in molecular simulations to perform structure-based virtual screening. SMASH works in interactive console mode, organizes calculations into user-defined projects, and can take proteins in PDB or PDBQT formats and ligands in three-dimensional SDF or MOL2 single-molecule forms. The source code is free and available to everyone. The current version is based on our previous UAM-Ixachi [5] workflow for Linux platforms.

SMASH combines CPU- and GPU-based methods into a single workflow to speed up the execution of long screening campaigns on local machines. The tested implementation worked with ×86–64 CPUs that support AVX2 extensions, as well as AMD, NVIDIA, and Intel graphics hardware. However, the level of compatibility depended on the specific docking engine and graphics stack used, which can vary significantly in terms of performance and features across different hardware configurations. We recommend at least 8 GB of RAM and at least 20 GB of free disk space. The exact amount of storage needed will depend on the size of the project. The current automatic installer was made for Ubuntu with APT package management. Table 1 shows the main differences between SMASH and UAM-Ixachi. Compared with UAM-Ixachi, SMASH represents an expanded workflow and a measurable methodological improvement.

**Table 1:** Comparison of the features in SMASH versus UAM-Ixachi

Characteristics	Ixachi	SMASH
Binding site identification method	Crystallographic ligand based	Crystallographic ligand and machine learning prediction
Binding site automatic parameters	Center only	Center and size
Atoms with alternative location treatment	Best occupancy	Best occupancy and intramolecular consistency
Protein titration states method	AutoDock Tools	PROPKA based
Docking engine	CPU based	GPU based
Scoring functions	Energetic	Energetic and machine learning based
Clustering	RMSD based	K-means based

**Fig. 1:**
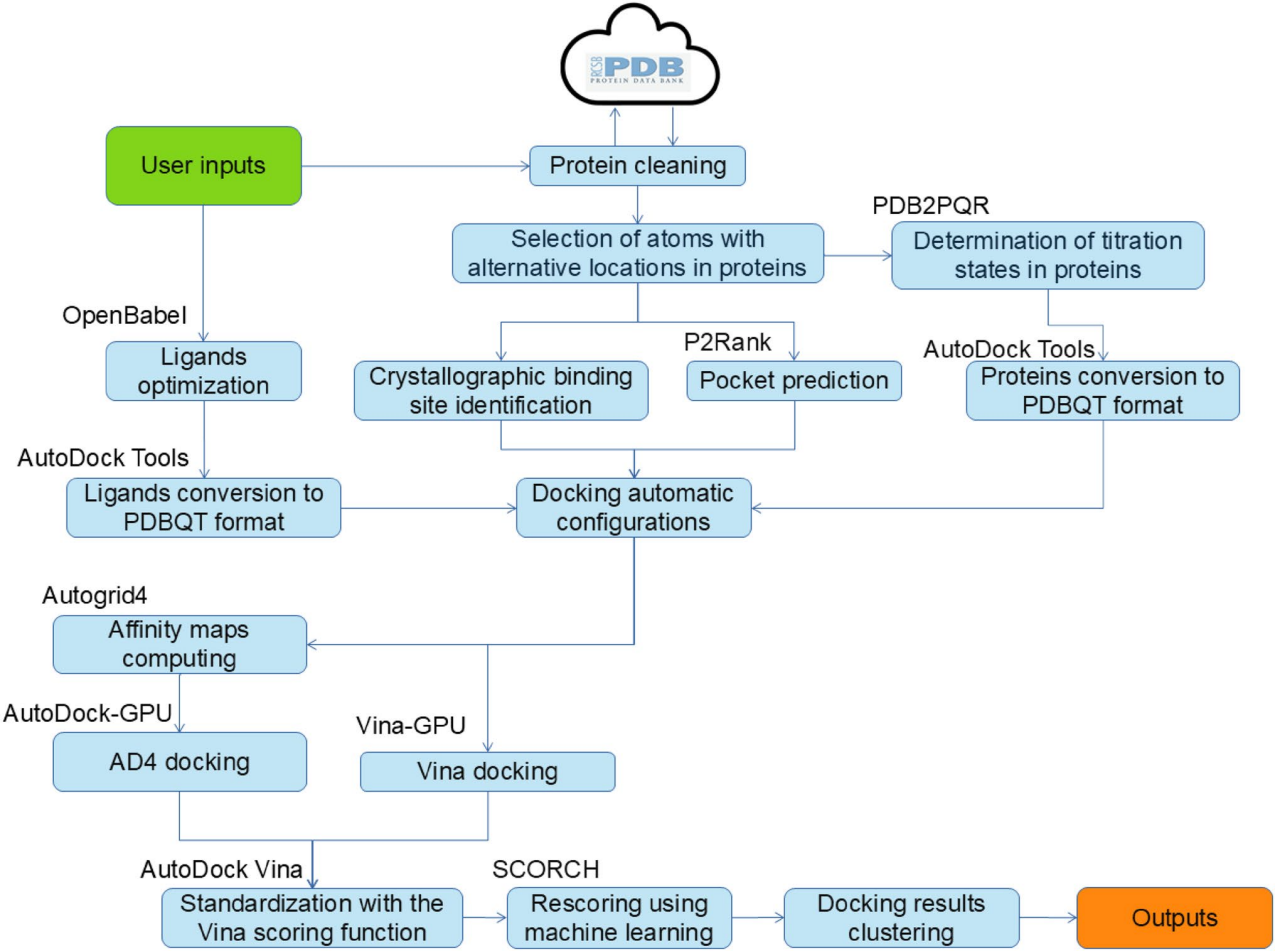
SMASH workflow. The tool used to solve each step is specified above it. Steps where this is not indicated are handled by SMASH

The process includes preparing receptors, identifying binding sites, preparing ligands, docking, rescoring, clustering, and generating summarized results. The output files include docking poses in PDBQT format, CSV files with docking and rescoring descriptors, clustering summaries, and graphs of the clustered results. Figure 1 shows the whole workflow.

### Automated receptor preparation

In SMASH’s automated receptor-preparation mode, the user enters PDB (Protein Data Bank) identifiers instead of modified structure files. This method was chosen because it relies on some PDB information, such as HETNAM and CONECT sections, to analyze receptors and identify ligand-binding sites. These parts are often changed or deleted by protein editing programs, leading to incomplete information that may affect the identification of binding sites. In automatic mode, SMASH retrieves the complete structure from the Protein Data Bank and examines the HETNAM, ATOM, HETATM, and CONECT sections to identify non-protein ligands covalently bound to the receptor.

This automated process removes water molecules, free ions and non-standard amino acids that are not covalently bonded to the protein. SMASH was designed this way because the importance of non-protein components (water, ions, lipids, carbohydrates, and crystallization additives) varies widely across systems and cannot be accurately assessed by the current automated process. Because of this, SMASH can handle manually curated receptor files in PDBQT format, allowing users to keep certain non-protein elements or to clearly define how they prepare their receptors.

For amino acid residue atoms with alternative atomic locations, SMASH keeps the atoms with the highest occupancy and discards the others. To improve structural consistency, the chosen positions are checked to ensure they are consistent with the remaining atoms. SMASH uses PROPKA [6] via PDB2PQR [7], a software tool that prepares protein structures for electrostatics calculations, to automatically assign titration states. This phase can restore a small number of missing heavy atoms, but it cannot bring back large areas that are missing, like loops. Protein preparation in the current version of SMASH follows a rigid-receptor docking framework; flexible-receptor docking is not implemented in the automated workflow.

### Binding-site identification and box generation

SMASH uses two different methods to find possible binding sites. The process first finds free ligands when co-crystallized ligands are available. It does this by using data from the PDB structure’s HETNAM, ATOM, HETATM, and CONECT records. The algorithm defines a free ligand as a group of atoms that has a residue designation from HETNAM, shares the same residue serial number, is not on a user-defined blacklist, and does not have CONECT records that show covalent attachment to the protein. The default blacklist includes compounds that are often found in PDB structures but are not usually pharmacologically important ligands.

**Fig. 2:**
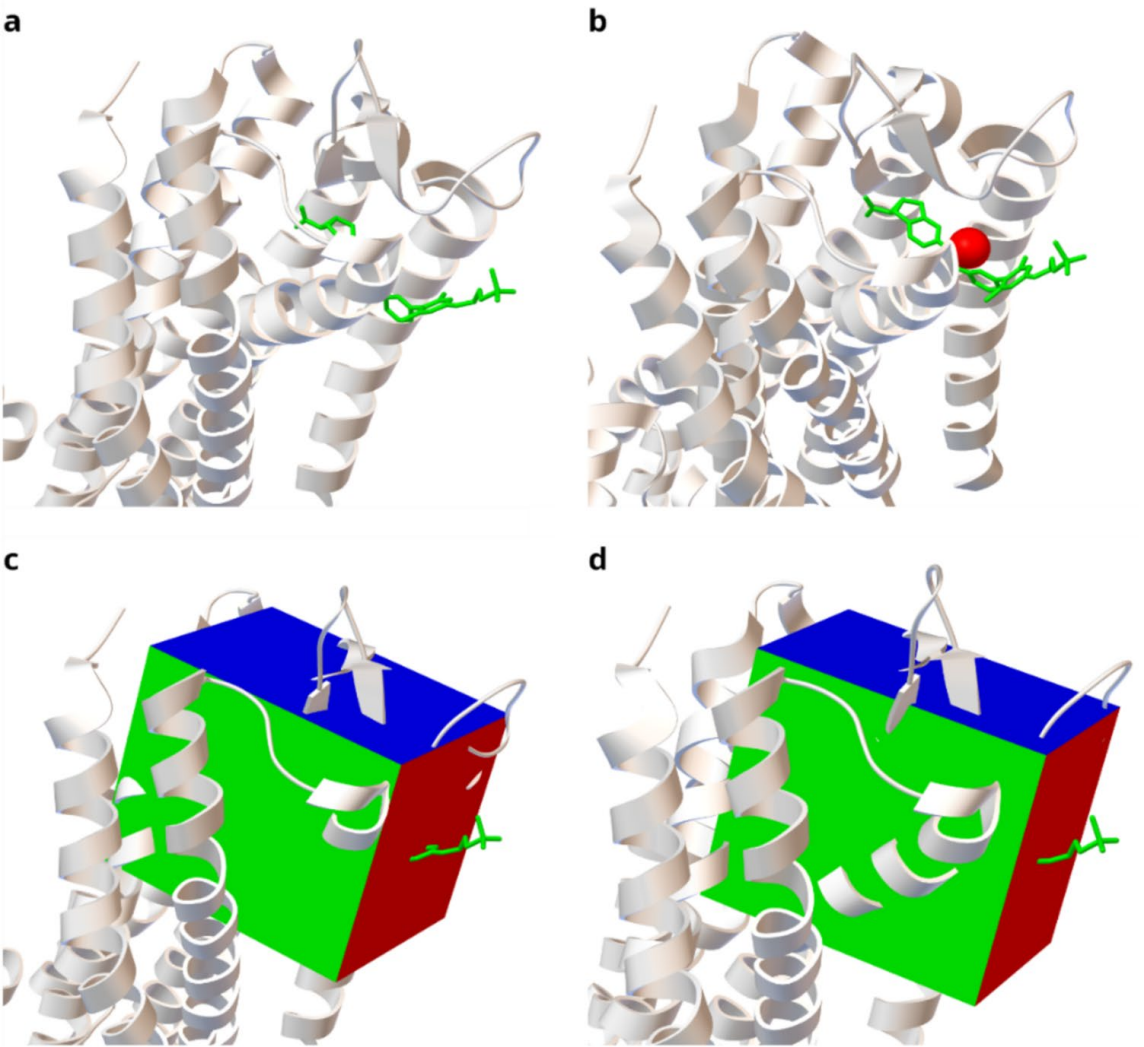
Illustration of automated methods for determining simulation boxes. The GPR40 receptor (PDB: 5TZR) is represented in complex with MK-8666 (green ligand). **a** Reference illustration of the complex. **b** Center of the simulation box (red sphere) calculated by UAM-Ixachi with the complete ligand. **c** Box calculated by SMASH based on amino acid residues surrounding the ligand. **d** Box based on P2Rank prediction

When SMASH finds a free ligand, it also finds all the amino acid residue atoms within 5 Å of each ligand atom and puts together the names of the amino acid residues. To find the minimum and maximum values along each axis, the coordinates of all the atoms that are part of those amino acid residues are used. From these values, the dimensions and the center of the simulation box are automatically calculated. This method was used to improve automatic box definition, especially when the crystallographic ligand is in an unusual position relative to the protein, as shown by the GPR40 example in Fig. 2. This procedure improves the identification of the binding site that arises from the unusual binding of the ligand and avoids misinterpretation of the search space.

If no suitable co-crystallized ligand is available, SMASH uses P2Rank [8] to estimate where the binding pockets might be. This lets the workflow examine orthosteric, allosteric, and other important pockets. By default, all experimental ligands provided by users are linked to all discovered binding sites for docking simulations, but users can disable ligand-site links that they do not want to run. P2Rank was selected because it is open-source, locally executable, relatively fast, and readily integrable into an automated workflow designed for local hardware. These practical considerations were central to the design goals of SMASH. In addition, we have found P2Rank effective at identifying known pockets. Furthermore, the software is very easy to implement in an offline workflow.

### Ligand preparation and docking workflow

Open Babel [9] optimizes ligands by assigning protonation states at pH 7.4 and using the MMFF94 force field to improve geometry. After that, the AutoDockTools scripts prepare_receptor4.py and prepare_ligand4.py [10] are used to convert receptors and ligands into PDBQT format.

SMASH runs docking on local hardware very fast by combining two GPU-accelerated docking engines: AutoDock-GPU [11] and Vina-GPU [12]. In its current implementation, SMASH uses a single GPU device, although CPU/GPU parallelism is exploited within the workflow. These tools calculate poses using the AD4 and Vina force fields, respectively. AutoGrid4 [10] was added to generate the affinity maps needed for AD4-based docking based on the types of atoms in the user-provided molecules. When both force fields are on, SMASH makes 100 poses for each ligand-site pairing with each docking engine. This means that there are 200 poses for each ligand-site pairing.

The workflow kept both docking engines because they used different methods to generate poses within the same automated framework. The current approach does not assume that one engine is better than all the others. Instead, it tests different poses under two different docking formulations. AutoDock Vina [13] operates in score-only mode, allowing poses to be compared later. This means that all poses are scored on the same scale.

### Rescoring, clustering, and result prioritization

The machine-learning-based scoring system SCORCH [14] is used to rate all poses, along with the Vina score. In this study, SCORCH was used as an additional way to prioritize, rather than replacing the Vina score. The goal of SMASH is to provide an additional measure of pose quality, along with a certainty value that can be used to sort results later.

SMASH uses a K-means clustering algorithm to automatically sort findings [15, 16]. It does this by using CSV files containing information on the ligand’s identity, docking energy, SCORCH score, and SCORCH certainty. In this work, the objective of clustering was not to capture geometric similarity, but to identify sets of solutions with similar energetic behavior and reliability. There are three descriptors that define the clustering space: energy, score, and certainty. Different clustering setups with 2 to 10 clusters are examined, and the silhouette coefficient indicates which is best in the defined descriptor space [17]. After figuring out how many clusters there should be, the centroid of each cluster is found, and the point closest to the centroid is chosen as the most representative member [18]. The descriptors are retained in their native scales, as no explicit normalization is applied. In this framework, docking energy serves as the primary discriminative axis, reflecting binding affinity, while SCORCH-derived metrics, bounded within range [0, 1], provide complementary information on structural plausibility and pose reliability. SMASH saves the results as separate CSV files and three-dimensional graphs. There is also a summary file (global_clusters.csv) that combines the clustering data for each protein, binding site, and force field combination [19]. K-means is run with a pre-set random seed (random_state = 0) to ensure consistency [20]. The similarity measure is Euclidean distance. In the current version of the workflow, the three clustering descriptors were not changed or normalized in any other way.

SMASH generates organized directories containing all docking poses in PDBQT format. The docking-power analyses shown used an internal F-factor based on the Vina and SCORCH scores to prioritize clusters. This factor was intended only as a useful guide for choosing clusters in the current test; it was not meant to be a new, reliable way to score results. The specific equation used in the analyses should be written down here as Eq. (1).

$$F= \left\{\begin{array}{lc}0, V>0\\ \left|VS\right|, V\le 0\end{array}\right.$$

where *V* is the Vina score and *S* is the SCORCH score.

### Validation datasets and computational setup

We evaluated SMASH based on its docking power, screening power, speed, and compatibility. Docking-power assessments were conceptually similar to those described by Su et al. [21]. and entailed re-docking ligands corresponding to the co-crystallized ligands of protein–ligand complexes from the PDB to assess crystallographic pose reproduction through root mean square deviation (RMSD). We assembled a set of 14 protein–ligand complexes with resolutions better than 3 Å and co-crystallized ligands suitable for these experiments (Table 2). A single binding site was selected for each structure based on the evaluation of the crystallographic orientation utilizing SCORCH and Vina. Simulations were performed at crystallographic ligand-binding sites, whereas P2Rank-based sites were set disabled. Ligands were obtained from PubChem when available or from the Protein Data Bank in an undocked conformation. All of SMASH’s standard automatic functions were used, including removing free ions and water molecules. Using DockRMSD [22], we calculated RMSD values and selected the representative conformation from the cluster centroids using the F-factor criterion we discussed.

**Table 2:** Parameters of the complexes chosen for the docking power test. NR is for “non-receptor.” The first 8 complexes were taken from the set assembled by Buccheri and Rescifina [23]

Class	Identity	PDB ID	Resolution (Å)	Bound ligand	Native pose Vina ΔG (kcal/mol)	Native pose SCORCH score
Hydrolase	Acetylcholinesterase	6O4W	2.35	(R)-donepezil	−11.53	0.45
NR tyrosine kinase	Tyrosine-protein kinase ABL2	2XYN	2.81	Tozasertib	−9.95	0.47
Metalloenzyme	Carbonic anhydrase II	4HT2	1.45	V50	−7.12	0.10
Protease	Beta-secretase 1	4DJW	1.9	Q27451162	−8.02	0.65
Ser/Thr Kinase	Cyclin-dependent kinase 2	1KE9	2	LSF	−7.95	0.66
GPCR	Adenosine A2a receptor	5OLH	2.6	Vipadenant	−9.91	0.15
Chaperone	HSP90α	4O09	1.96	2R6	−12.12	0.80
Histone deacetylase	HDAC 6	5EDU	2.79	Trichostatin A	−5.41	0.85
Transcription factor	ER alfa	5TOA	2.5	Estradiol	−9.60	0.80
Binding protein	Streptadivin	1STP	2.6	Biotin	−6.27	0.92
Transferase	Glutathione-S-transferase	2C3Q	1.85	S-hexylglutathione	−6.35	0.28
Oxidoreductase	Vascular adhesion protein-1	4BTX	2.78	WF8	−8.39	0.02
Ligase	Acyl-CoA synthetase	2WD9	2.6	Dexibuprofen	−5.60	0.20
Ion channel	Torpedo acetylcholine receptor	7SMR	2.77	Carbamoylcholine	−3.78	0.88

We used active and decoy ligands from the DUD-E [24] database to test screening efficacy and time. In this benchmark, 2912 ligands were docked to adipocyte fatty acid binding protein (FABP; PDB: 2NNQ). The tests were performed on a desktop computer running Ubuntu 24.04.2. It had an AMD Ryzen 5 7600X CPU, an NVIDIA GeForce RTX 4070 SUPER GPU, and 32 GB of RAM.

We have not included head-to-head benchmarks comparing UAM-Ixachi with SMASH because both versions are designed to run on consumer-grade hardware. Conducting equivalent tests on UAM-Ixachi would be impractical, as this version relies on CPUs and would require an excessive amount of time to complete such evaluations.

We used a benchmark set included in SMASH to test compatibility on three different hardware setups: (i) a desktop computer with an AMD Ryzen 5 7600X CPU and NVIDIA GeForce RTX 4070 SUPER GPU, (ii) a laptop with an Intel Core i3 1215U CPU and integrated Intel UHD graphics, and (iii) a desktop computer with an AMD Ryzen 5 7600X CPU and AMD Radeon RX6600 GPU. We tested Ubuntu versions 24.04.2 and 22.04.5 on these systems.

## Results

**Fig. 3:**
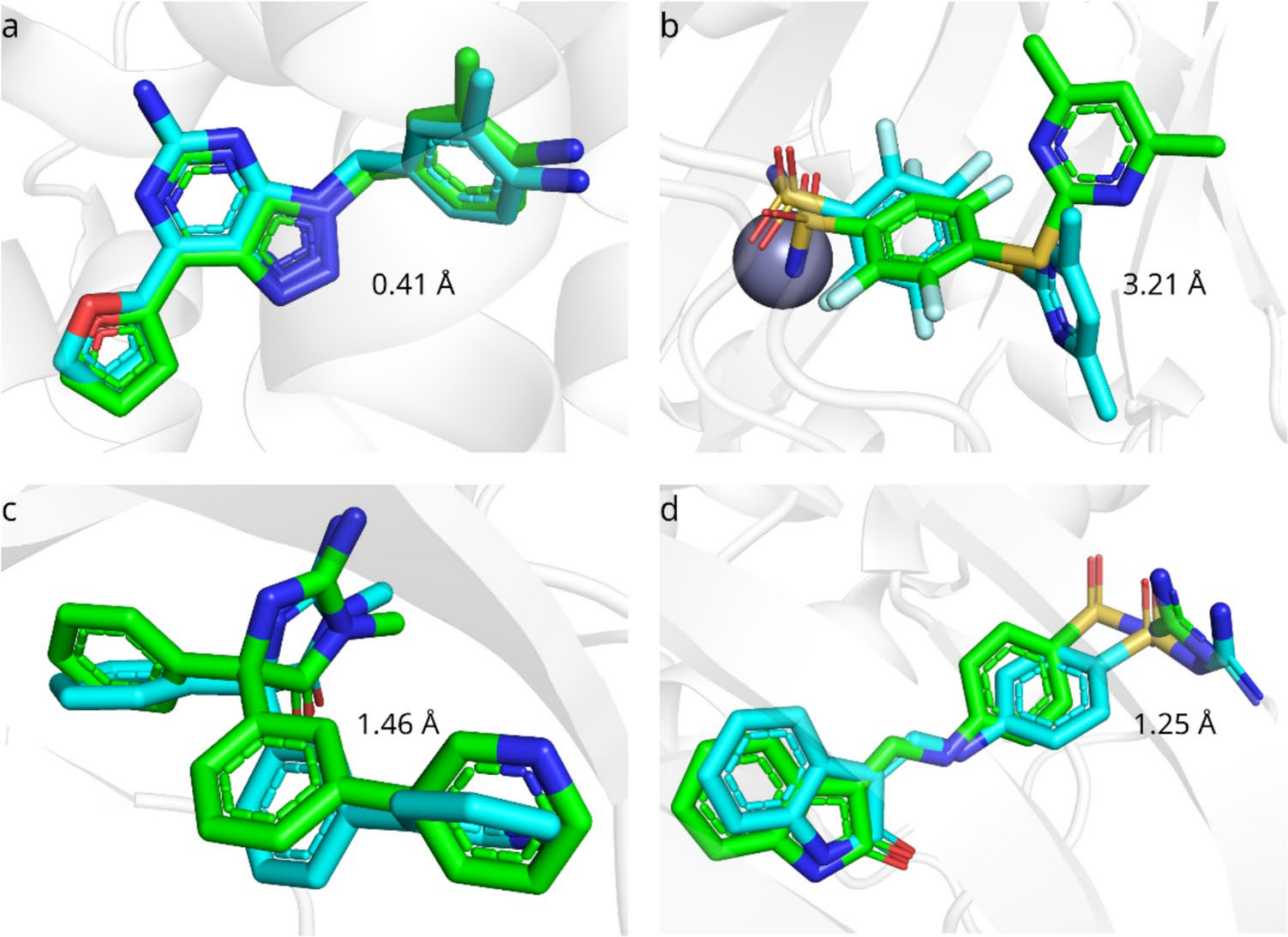
Selection of overlay comparisons between the native poses and the SMASH-simulated poses; the RMSD value is indicated next to each pair of overlaid molecules. The green carbon chain corresponds to the native pose. **a**, **b** The best and the worst RMSD of the test, respectively: Adenosine A2a receptor in complex with Vipadenant (PDB: 5OLH) and Carbonic anhydrase II in complex with V50 (PDB: 4HT2). **c**, **d** Central RMSD values, respectively: Beta-secretase 1 in complex with Q27451162 (PDB: 4DJW) and Cyclin-dependent kinase 2 in complex with LSF (PDB: 1KE9)

After a visual inspection of the molecules automatically prepared by SMASH, no structural abnormalities were found. SMASH reproduced the crystallographic pose in 11/14 complexes (Table 3), with an RMSD of less than 2 Å; the worst RMSD was 3.21, and two other cases had RMSD values barely exceeding 2 Å. A selection of these results is illustrated in Fig. 3.

**Table 3:** Results of the docking power test. RMSD values are given in Å and energy in kcal/mol. The site name is a PDB-like denomination: ligand PDB ID, PDB chain, and serial number. The results shown here were selected by the best F-factor of each simulation

Identity	PDB ID	Site name	Ligand	PubChem ligand CID	Vina energy	SCORCH score	RMSD
Acetylcholinesterase	6O4W	E20 B 604	(R)-donepezil	1,150,567	−11.00	0.76	1.70
Tyrosine-protein kinase ABL2	2XYN	VX6 B 547	Tozasertib	5,494,449	−10.76	0.52	0.94
Carbonic anhydrase II	4HT2	V50 D 302	V50	71,299,336	−7.41	0.21	3.21
Beta-secretase 1	4DJW	0KP A 501	Q27451162	70685E6	−9.02	0.90	1.46
Cyclin-dependent kinase 2	1KE9	LS5 A 299	LSF	-	−9.47	0.77	1.25
Adenosine A2a receptor	5OLH	9XT A 1201	Vipadenant	21,874,557	−8.93	0.24	0.41
HSP90α	4O09	2R6 A 4000	2R6	73,437,615	−11.88	0.85	1.00
HDAC 6	5EDU	TSN A 904	Trichostatin A	444,732	−7.32	0.74	2.06
ER alfa	5TOA	EST A 601	Estradiol	5757	−9.91	0.88	0.66
Streptadivin	1STP	BTN A 300	Biotin	171,548	−7.68	0.98	0.64
Glutathione-S-transferase	2C3Q	GTX D 301	S-hexylglutathione	-	−6.70	0.37	1.70
Vascular adhesion protein-1	4BTX	WF8 B 2000	WF8	-	−7.24	0.15	1.74
Acyl-CoA synthetase	2WD9	IBP A 1570	Dexibuprofen	39,912	−7.00	0.59	2.07
Torpedo acetylcholine receptor	7SMR	CCE D 601	Carbamoylcholine	2551	−4.50	0.91	0.58

**Fig. 4:**
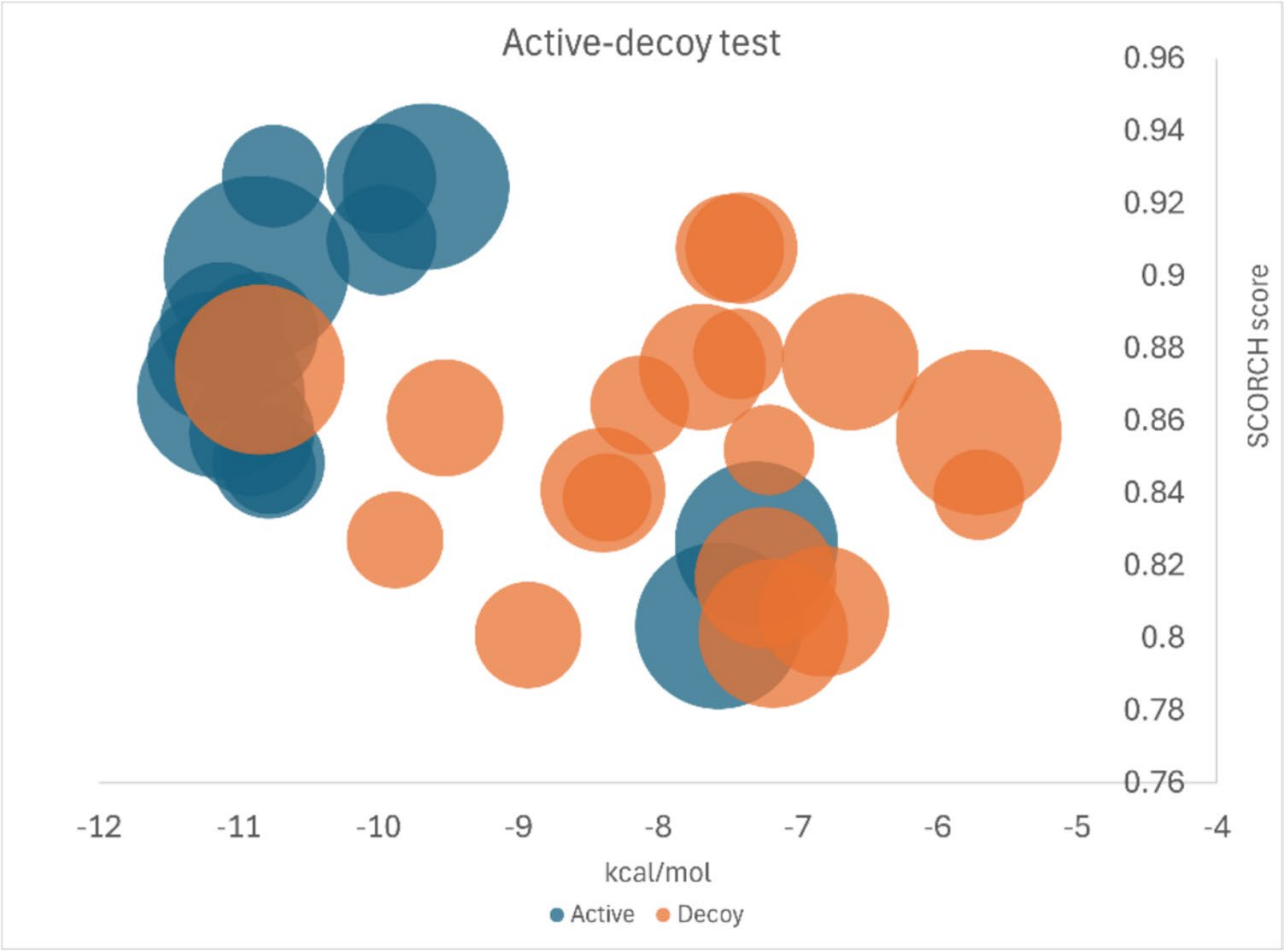
Bubble plot showing the results from the screening power test of active and decoy ligands on the FABP protein (PDB: 2NNQ). The diameter corresponds to the cluster population; the biggest diameter is 100 poses

Figure 4 represents the results from the screening power test that meet the following conditions: SCORCH score of 0.8 or higher, SCORCH certainty of 0.8, and cluster size of 20 or higher. Only clusters with the best F-factor of each simulation were eligible for these results. The active ligands tended toward the region with the best scores in the graph (upper left corner), while the decoys showed clusters in the less favored part (lower right corner).

The timing test yielded the following results: Preparation of 2912 ligands took 9 min; docking simulations with AutoDock-GPU took 56 min; Vina-GPU simulations took 3.32 h; rescoring energies with AutoDock Vina (for poses generated by AutoDock-GPU only) took 6.26 h; calculating the SCORCH score took 11.54 h; and the clustering process took 14 min. The total time was 24.13 h, according to the SMASH log files (timings of minor procedures with negligible time and steps with fuzzy boundaries were omitted). The average processing time for each ligand was approximately 30 s.

SMASH compatibility testing showed that it is stable on the PC configurations described in the methodology, except for the one that includes an integrated GPU with Intel UHD graphics. Vina-GPU did not work for this due to incompatibility with the Intel GPU OpenCL version. The program endured the failure and continued running only AutoDock-GPU.

## Discussion

SMASH was designed as a single local workflow that automates receptor preparation, binding-site identification, ligand preparation, docking, rescoring, clustering, and reporting in a single Linux-based environment. SMASH is distributed for a defined Linux environment to improve reproducibility and simplify dependency handling for end users. The main goal is to make it easier to start a docking campaign by reducing the number of steps that need to be done manually and enabling researchers to use local computers, such as basic laptops and desktops. In this situation, SMASH improves the previous UAM-Ixachi workflow by adding automatic box-size estimation, machine-learning-based pocket prediction, GPU-accelerated docking, machine-learning-based rescoring, and automatic result clustering.

The docking power analyses show that SMASH can automatically reproduce many crystallographic poses, with RMSD values below 2 Å in 11 of the 14 complexes tested. The outliers in this benchmark show that automated preparation does not guarantee that all targets will reproduce the ligand pose with the same level of accuracy. This is especially important for systems where interactions are heavily affected by parts removed in automatic mode, such as free ions or other structural elements. The results should be viewed as indicative of the approach’s potential efficacy in actual docking campaigns, rather than as validation of consistently superior predictive accuracy across all receptor classes.

The addition of both AutoDock-GPU and Vina-GPU was based on how well they work together, not on the idea that both engines are always needed. We have implemented two methods for generating poses that sample possible poses across different docking engines and then compare them on a single Vina scoring scale. When we tested AutoDock-GPU and Vina-GPU for speed and compatibility, AutoDock-GPU was much faster at generating poses and showed better functional stability across the hardware configurations we tested. Vina-GPU, on the other hand, offered a different approach to generating poses within the same automated framework by using a different search algorithm.

In addition, SCORCH played a secondary role in our research. It was advantageous during pose selection when the Vina energy alone could not clearly identify the posture that best matched the crystallographic reference. It also made it easier to distinguish between poses with similar energies and different RMSD values. However, this extra information requires significant computing power because SCORCH accounted for a large portion of the total wall time in the timing benchmark. Therefore, SCORCH should be viewed as a tool for prioritizing ligand poses within the workflow, not as a clear improvement over traditional energy-based scoring.

Clustering analysis revealed a structured organization of docking results, where groups of poses are primarily differentiated by their energetic profiles, while machine learning scoring metrics refine selection within each group. This behavior indicates that docking energy is the main discriminative factor, whereas SCORCH-derived metrics provide complementary information on structural plausibility and pose reliability.

SCORCH proved useful in distinguishing between poses with similar energies but differing structural quality, reducing the likelihood of selecting energetically favorable yet geometrically inaccurate solutions. This highlights the importance of incorporating complementary scoring approaches beyond purely energetic criteria, especially when energy alone is insufficient to identify near-native conformations. Overall, the clustering results reflect an energy-driven organization of the solution space, in which SCORCH-derived metrics enhance the robustness and reliability of pose selection.

The screening benchmark shows that the workflow can distinguish active molecules from decoys in some cases. This is because active ligands usually occupy better spots on the score plot than decoys. This study was limited to a single DUD-E target and was visualized after the implementation of additional filters based on SCORCH score, SCORCH certainty, cluster size, and cluster selection. As a result, these results should be seen with caution as a real-world example of how workflow works, not as a clear example of how well screening works.

The clustering variables were docking energy, SCORCH score, and SCORCH certainty. The representative pose was the one closest to the cluster center, not the one with the highest score. This choice gives greater weight to a position representative of the cluster, reducing the possibility of selecting an outlier. The current implementation is based on the Euclidean distance between the raw values of the three variables instead of normalized values. Because the range of docking energies exceeds the range of the SCORCH variables, this gives more weight to energy in the clustering. The F-factor was used only as an internal guideline for selecting clusters in the documented experiments, and it should not be considered a new scoring method.

Another limitation of the automated process is that receptor preparation remains context dependent. The automated mode is useful for making large projects more consistent, but some systems still require expert work. This includes decisions about solvent molecules, free ions, metalloproteins, unusual cofactors, manually assigned protonation states, or large gaps in experimental structures. In these cases, it is very important to prepare the receptors by hand and to carefully examine the results. Therefore, we do not see SMASH as a replacement for expert analysis; instead, we see it as a tool that can reduce preparation time and enable first-screening campaigns to be started while still allowing for personal intervention when needed.

The timing results show that moderate virtual screening campaigns can be run on local hardware within a time frame useful in practice. The FABP benchmark showed that it took 24.13 h to process all 2912 ligands, with each ligand taking about 30 s on average. This performance profile is one of SMASH’s main practical contributions. Its main goal is not to use supercomputers for large-scale screening but to use relatively new, cheap, or mid-range local computers for thorough structure-based virtual screening.

The results show that SMASH is a viable automated workflow for local docking and moderate-scale virtual screening, especially when it is important for the user to be able to use it easily, run it locally, and combine different preparation and scoring processes. The current validation must be understood within its actual parameters: the reported tests illustrate workflow integration, practical runtime, and acceptable docking behavior in specific benchmarks, yet they do not represent a comprehensive validation of all incorporated third-party engines across all potential target classes.

There are other excellent, free, and user-friendly tools, such as EasyDoc [25], or platforms designed for very large-scale projects, like VirtualFlow [26], which require execution on supercomputers. Our goal is likewise to provide a free and intuitive tool for virtual screening; however, we place equal emphasis on optimizing performance for local, even modest, computational resources. This approach democratizes access to computational simulations, enabling researchers without supercomputers to carry out meaningful studies. In this context, SMASH allows to work efficiently on relatively modern, low-cost, or mid-range desktop and laptop computers, delivering shorter calculation times while maintaining accuracy in the results.

## Conclusions

SMASH is the result of our group’s ongoing efforts to develop computational prediction tools with pharmacological applications. It is designed to be accessible, both economically and technically, while providing results with predictive value. We are committed to maintaining support for SMASH and to continuously improving its capabilities, with plans to offer an online service and to release a version that scales efficiently across computing clusters. Future developments will focus on enhancing the algorithms to further reduce computational time, expanding compatibility with diverse platforms, and ensuring broader support for a wide range of hardware and operating systems.

## Supplementary Information

Below is the link to the electronic supplementary material.

ESM 1 (DOCX 51.5 KB)

## Data Availability

The tool installer can be downloaded from https://smashreleases.z13.web.core.windows.net/ along with the user manual
