# Supplementary material for: SMASH: Screening Molecules Accurately on Small Hardware. Fast, user-friendly, enhanced with a machine learning virtual screening tool
Source: J Mol Model. 2026 Jul 4;32(8):257. doi: 10.1007/s00894-026-06837-x (PMC13332966; doi:10.1007/s00894-026-06837-x)
Supplement: Supplementary file 1 — (DOCX 51.5 KB) [file 894_2026_6837_MOESM1_ESM.doc]

**SMASH: Screening Molecules Accurately on Small Hardware. Fast, user-friendly, enhanced with machine learning virtual screening tool**

Supplementary data

Table S1. SMASH default operating parameters

| Application | Parameter | Value |
| --- | --- | --- |
| PDB2PQR | Protein pH | 7.4 |
| PDB2PQR | Method | propka |
| PDB2PQR | Force field | CHARMM |
| OpenBabel | Ligand pH | 7.4 |
| OpenBabel | Ligand minimization iterations | 3200 |
| OpenBabel | Force field | MMFF94 |
| SMASH | Min P2Rank pocket score | 0.5 |
| SMASH | Max CPU parallelism | 100% |
| SMASH | Free ions delete | True |
| SMASH | Water delete | True |
| SMASH | Scikit-learn/NumPy Clustering score normalization | True |
| SMASH | Minimum number for clusters to explore | 2 |
| SMASH | Maximum number for clusters to explore | 10 |
| AutoDock-GPU | Asymptotic heuristics number evals limit | 1.2*10^7^ |
| AutoDock-GPU | Auto-stop testing frequency | 5 |
| AutoDock-GPU | Lamarckian genetic algorithm (LGA) runs | 100 |
| AutoDock-GPU | Max. score evaluations per LGA run | 2.5*10^7^ |
| AutoDock-GPU | Max generations per LGA run | 4.2*10^4^ |
| AutoDock-GPU | Max. Local-search iterations | 300 |
| AutoDock-GPU | Population size | 150 |
| AutoDock-GPU | Mutation rate | 2 (%) |
| AutoDock-GPU | Crossover rate | 80 (%) |
| AutoDock-GPU | Local-search rate | 100 (%) |
| AutoDock-GPU | Tournament (selection) rate | 60 (%) |
| AutoDock-GPU | Maximum LGA movement delta | 6 (Å) |
| AutoDock-GPU | Maximum LGA angle delta | 90 (°) |
| AutoDock-GPU | Solis-Wets lower bound of rho parameter | 0.01 |
| AutoDock-GPU | Solis-Wets movement delta | 2 (Å) |
| AutoDock-GPU | Solis-Wets angle delta | 75 (°) |
| AutoDock-GPU | Solis-Wets cons. success/failure limit to adjust rho | 4 |
| AutoDock-GPU | Auto-stop energy standard deviation tolerance | 0.15 (kcal/mol) |
| AutoDock-GPU | Smoothing parameter for vdW interactions | 0.5 (Å) |
| AutoDock-GPU | Min. electrostatic potential distance | 0.01 (Å) |
| SMASH | Vina-GPU docked ligand pose count | 100 |
